# Techniques for Respiratory Motion-Resolved Magnetic Resonance Imaging of the Chest in Children with Spinal or Chest Deformities: A Comprehensive Overview

**DOI:** 10.3390/jcm14092916

**Published:** 2025-04-23

**Authors:** Paula Arias-Martínez, Peter P. G. Lafranca, Firdaus A. A. Mohamed Hoesein, Koen Vincken, Tom P. C. Schlösser

**Affiliations:** University Medical Center Utrecht, 3584 CX Utrecht, The Netherlands; paularimart@gmail.com (P.A.-M.); p.p.g.lafranca-2@umcutrecht.nl (P.P.G.L.); f.a.a.mohamedhoesein@umcutrecht.nl (F.A.A.M.H.); k.vincken@umcutrecht.nl (K.V.)

**Keywords:** MRI, motion, respiration, scoliosis, thoracic deformity

## Abstract

Quantification of the severity of chest wall deformation in children with spinal deformities is essential for understanding the effects on trunk appearance and cardiopulmonary function. Magnetic resonance imaging (MRI) is particularly valuable for this purpose, as it does not employ ionizing radiation and can provide three-dimensional (3D) imaging of thoracic anatomy. Acquiring sufficient quality images of the chest wall, lungs and airways at key stages of the respiratory cycle, such as end-inspiratory or expiratory phase, is crucial for accurately assessing chest wall deformation and pulmonary function and mechanics. Regarding image quality, low proton density and short relaxation times of the lung tissues result in poor quality images, and long acquisition times result in blurring caused by respiratory and cardiac motion. This overview summarizes strategies developed to address the inherent challenges of visualization of lung tissue and respiratory motion in MRI acquisition of the chest of pediatric patients with spinal deformities. An overview of the main methods for motion-resolved image acquisition and measurement of chest wall motion and thoracic volumes is presented and discussed. It is concluded that despite the development of multiple techniques and diverse strategies for obtaining high-quality, motion-resolved chest MRI, further validation of these methods is required before their implementation in clinics for routine evaluation of chest deformation in pediatric spinal deformity patients.

## 1. Introduction

Adolescent idiopathic scoliosis (AIS) is a three-dimensional deformity of the spine and trunk with a prevalence of 1–3% [1]. Besides the spinal deformity and related back pain, AIS can lead to chest wall pain, changes in trunk appearance, as well as pulmonary impairment, mostly due to associated chest wall deformation [2]. The pulmonary impairment typically involves restrictive pulmonary capacity due to decreased lung volumes, restricted chest wall motion and altered mechanics of the diaphragm and chest wall muscles [3]. Furthermore, due to the intrusion of the spine into the chest in AIS, the main airways on the convex side of the spinal curve can become compromised, resulting in an additional obstructive loss of pulmonary function [4]. In early-onset scoliosis (EOS), insufficient growth of the chest (also known as thoracic insufficiency syndrome) is also associated with insufficient lung development and increased mortality [5]. Functional respiratory tests, such as spirometry or plethysmography, have mainly been used for evaluating the short- and long-term effects of the chest deformation on pulmonary function in scoliosis patients [6]. As they require patient cooperation and do not reflect the other consequences of the chest wall deformation in AIS, instruments for evaluating the pathoanatomical effects of 3D chest deformation are of clinical value for this vulnerable patient population.

While various medical imaging techniques have been developed for evaluating in vivo morphological changes in the chest for diseases such as asthma, cystic fibrosis or tracheobronchomalacia, medical imaging techniques for quantification of respiratory mechanics and chest wall deformation in scoliosis patients have received less attention [7,8]. Three-dimensional imaging techniques can be particularly beneficial for assessing lung function and chest volume in patients with AIS or EOS associated with thoracic insufficiency syndrome (TIS). Notably, specific evaluations have been performed using computed tomography (CT) or 3D reconstructions of biplanar radiographs [9,10,11]. CT acquisitions in children, however, raise concerns about long-term effects of radiation exposure. This is especially important as a previous study showed a five-times-higher cancer prevalence in AIS patients [12]. Moreover, CT and biplanar radiograph acquisitions are generally static, where a dynamic assessment of the chest is probably more valuable [13]. Therefore, magnetic resonance imaging (MRI) is being explored as a radiation-free 3D imaging alternative, to cover dynamic assessments of chest wall deformation and respiratory mechanics.

The acquisition of clinically valuable chest MRIs presents multiple challenges in children with spinal deformities: the low proton density of lung parenchyma leads to a reduced signal-to-noise ratio (SNR), the air-tissue interfaces within the pulmonary tissue induce localized magnetic susceptibility gradients, increased signal dephasing and, thus, too short T2* relaxation times, and most importantly, cardiac and respiratory motions interfere with the prolonged imaging times of MRI [14,15]. To address these challenges, diverse MRI techniques have been investigated not only to control and reduce motion artifacts, but also to enable a better assessment of the chest wall and respiratory mechanics [15,16]. The aim of this overview is to provide a comprehensive overview of MRI techniques for controlling or tracking respiratory motion in chest MRI and discuss how these techniques can be utilized for determining dynamics of the chest wall, thoracic volumes and respiratory function in patients with spinal deformities.

## 2. Methods

### Search Strategy and Study Selection

Despite no specific guidelines or systematic methodologies being applied for this narrative review, the literature search was conducted with rigor in relevant, peer-reviewed, scientific databases. Moreover, clear inclusion and exclusion criteria were established.

The search strategy was designed to ensure as wide a range of studies on respiratory motion-resolved magnetic resonance imaging of the chest as possible. A search was conducted across PubMed, Google Scholar and ScienceDirect digital databases using the following words: “MRI” AND (“motion” OR “breathing” OR “respir*”) AND (“chest” OR “scoliosis” OR “lung”). First, all studies were screened on title and abstract. Afterwards, the remaining studies were screened on full text.

The selection process was guided by key elements of the Population–Concept–Context (PCC) framework, as recommended by the Joanna Briggs Institute (JBI) (North Adelaide, Australia) for structuring inclusion criteria [17]. Specifically, the Population consisted of individuals undergoing chest MRI, with special interest for studies including populations of patients with scoliosis or other thoracic deformities. The Concept focused on respiratory motion control techniques in MRI, methods for reconstructing the lungs along different breathing stages or quantification of thoracic volumes. The Context was centered on the measurement of lung function or estimation of respiratory signals. Research addressing respiratory motion with focus on cardiac MRI or other than the chest was excluded from the overview.

## 3. Results

In total, 75 studies were found and screened for this study on title and abstract. Of these, 52 studies were screened on full text, resulting in a total of 19 papers discussing motion control techniques and reconstruction of chest MRI on different breathing phases being included. Of these studies, eight also included analysis of pulmonary function and thoracic volumes in healthy individuals and/or patients with spinal deformities. Several studies acquired 2D dynamic images, thereby capturing the different breathing stages in a single slice location. Other studies produced static 3D images of the chest at specific breathing phases. Lastly, 4D images were also achieved by generating continuous 3D images throughout the complete breathing cycle. A summary of these articles is provided in Table A1 in the Appendix A.

### 3.1. Respiratory Motion-Resolving Techniques

The standard technique for compensating for respiratory movement is motion gating. This approach limits the acquisition of MR data to a specific motion phase. It can be achieved either prospectively, during acquisition; and/or after acquisition, during image reconstruction [18].

In lung MRI, controlling respiratory motion artifacts to obtain good quality diagnostic images can be achieved prospectively by synchronizing the acquisition process with the patient’s breathing. Breath-holding requires significant patient cooperation, which is not always feasible, especially with young children [14]. In addition, acquisition times are considerably limited by breath-holding capabilities [19].

External devices can be employed to synchronize image acquisition with breathing. Examples include the pneumotachograph, which records respiratory flow and volume signals, or the “MR-compatible active breathing control” device [20,21,22]. However, these methods entail a substantial technical effort and still rely on some degree of patient cooperation, such as wearing masks or mouthpieces.

**Figure 1:**
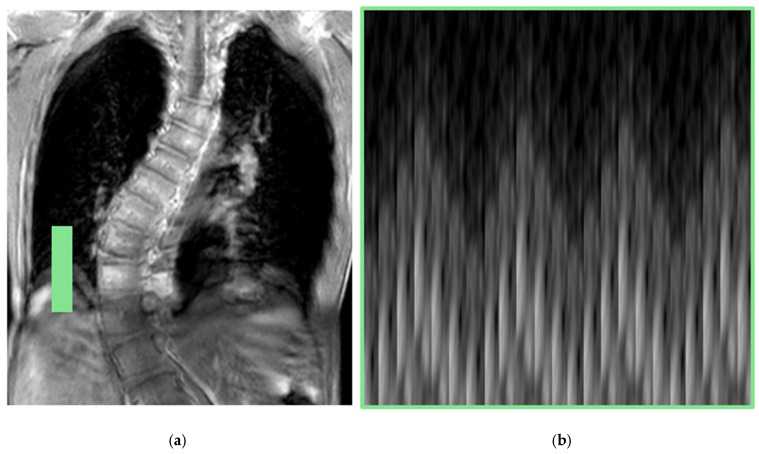
Navigator echo. (**a**) A thin field of view (green area) is excited over the diaphragm dome. (**b**) The respiratory waveform is reconstructed. This image does not represent a real navigator echo acquisition but is intended solely to illustrate the concept.

In free-breathing acquisitions, breathing motion can be tracked by measuring the changes in chest circumference using respiratory belts, or “pneumobelts”, which consist of an adjustable tube positioned around the chest [23]. Another method is by means of navigator echoes. These detect diaphragm position over time by applying an additional RF-pulse to a small field of view over this structure along the direction of maximum movement, thereby reconstructing the respiratory waveform [14,19,24]. This is illustrated in Figure 1. Navigator echoes present some disadvantages, as the additional RF-pulses, which are interleaved, may prolong the acquisition time and cause steady-state perturbations on some regions of the volume to be imaged that become saturated. Thus, the k-space center (DC) signal, which represents the total signal of the volume being acquired, has been used as well as a self-gating signal. The diaphragm’s motion alters the spin density within the excited slice, inducing variations in the DC signal. By placing a single coil near the diaphragm, respiratory-induced signal changes can be detected, enabling synchronization of data acquisition with breathing [19,25].

These strategies served as the basis for acquiring motion-resolved chest MR images at specific breathing phases or throughout the entire respiratory cycle. This enabled the generation of 2D, 3D and 4D pulmonary MR images, valuable for assessing pulmonary function in children with spinal deformities. The different kinds of imaging protocols and reconstruction methods for achieving these images are detailed below. Table 1 summarizes the different gating techniques employed by the articles included in this overview. Table 2 shows the types of images produced by the articles, along with the advantages and disadvantages of each type.

**Table 1:** Motion-gating techniques employed by the articles included in this overview.

Moment of Respiration Resolving	Gating Technique	Study
At acquisition	Breath-holding	Chu et al. [26]
		Chu et al. [27]
	Breathing with maximal inspiration/expiration	Plathow et al. [8]
		Kotani et al. [28]
	Synchronized image acquisition using flow and volume signals from external devices (e.g., pneumotachograph, MR-ABC)	Kondo et al. [21]
At image reconstruction	Motion-gated image reconstruction using chest circumference changes measured by respiratory belts	
	Estimation of respiratory waveform from navigator echoes	Wachinger et al. [29]
		Tibiletti et al. [25]
	Estimation of respiratory waveform from k-space center (DC) signal	Weick et al. [19]
		Feng et al. [16,30]
		Chen et al. [31]
		Higano et al. [15]
		Jiang et al. [32]
		Xu et al. [33]
		Miller et al. [34]
	Other advanced techniques that do not generate estimates of respiratory signal. Based on spatial and temporal similarity/continuity, or measurement of the motion vector field.	Baumgartner et al. [35]
		Tong et al. [36]
		Hao et al. [37]
		Sun et al. [38]

#### 3.1.1. Two-Dimensional MRI

Several studies carried out dynamic MRI investigations, using sequences such as fast spin echo, trueFISP, fast spoiled gradient-recalled echo and fast gradient-recalled echo [8,21,26,28].

**Figure 2:**
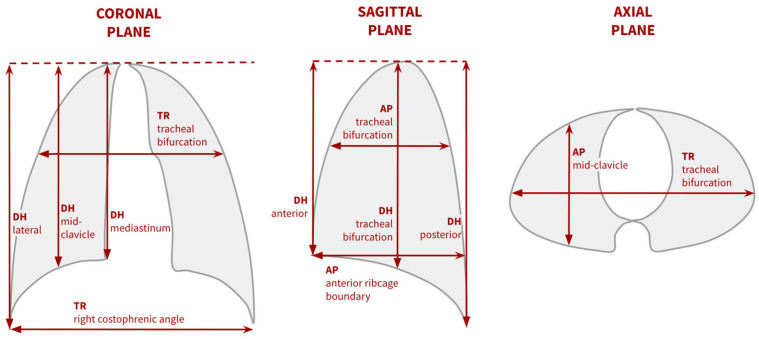
Main distances and diameters measured in dynamic MRI studies [8,21,26,28]. These are typically measured on full-inspiration and full-expiration images to estimate the chest and diaphragm motion by calculating the difference between both measurements. Abbreviations: DH = diaphragmatic height, AP = anteroposterior diameter, TR = transverse diameter.

Some analyzed respiratory-related movements of the chest wall and diaphragm in healthy subjects, in correlation with standard respiratory function tests. Representative diameters in 2D sagittal, axial and coronal slices (Figure 2) were measured separately at maximum inspiration and expiration. Thus, these acquisitions required patients to follow instructions on their respiration cycle. The differences between the diameters at each timepoint provided an estimate of the chest wall motion at different locations [8,21]. Additionally, cross-sectional areas were also measured in sagittal and axial planes, also at maximum inspiration and expiration. These measurements correlated with lung volume changes measured with pneumotachographs [21]. Strong and significant correlations were also found between MRI-calculated vital capacity (VC), estimated using a simplified model of the Barnhard–Loyd elliptical method, and spirometry VC [8,39]. Other studies investigated chest wall, diaphragmatic motion and lung volume in patients with AIS compared with normal controls [26,28]. Similarly to the previous studies, chest wall and diaphragm movements were estimated by measuring representative diameters in 2D slices at inspiration and expiration stages. Limited chest wall motion in the patient group, especially in the anteroposterior direction, was a distinguishing feature of respiratory impairment [28]. In addition, lung volumes were also measured from the MR images by adding cross-sectional areas in contiguous slices. A significant reduction was found in inspiratory and expiratory lung volumes in AIS patients with respect to healthy subjects [26]. Following these strategies, lung function was also assessed in MRI before and after posterior spinal fusion surgery in AIS patients, finding a significant increase in movement in the transverse direction, as well as in diaphragmatic motion after this procedure [27].

#### 3.1.2. Three-Dimensional MRI

##### Fast 3D Imaging: Motion-Resolved MRI Using DC Signal

**Figure 3:**
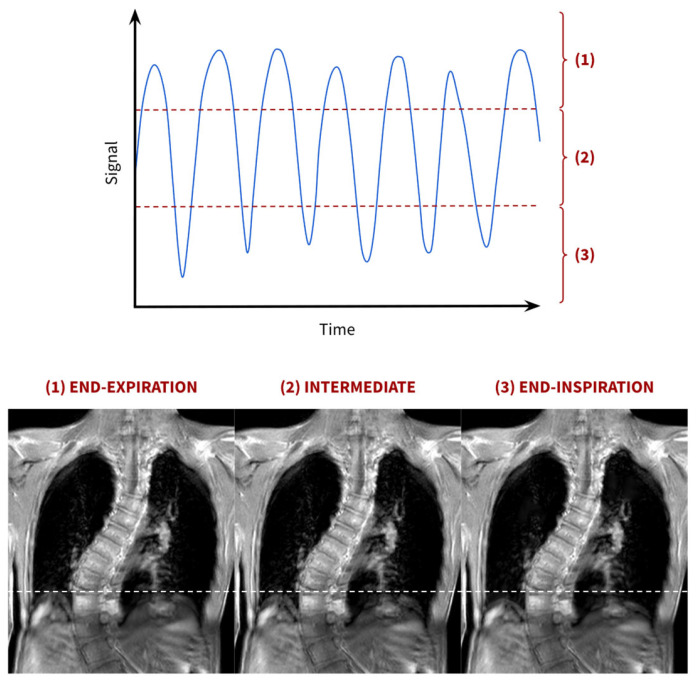
Reconstruction of images at different respiratory phases. Images corresponding to different respiratory phases can be reconstructed by selecting different threshold values on the DC signal respiratory waveform. The red dotted lines delimit the thresholds between the three breathing phases. The white dotted line provides a visual aid to compare the diaphragm position among the three respiratory phases. This figure does not depict a real DC signal waveform or reconstruction but is intended solely to illustrate the concept.

For the neuromuscular and early-onset scoliosis patient populations, free-breathing image acquisition is often practically the only viable option. Consequently, significant research has focused on employing fast MRI sequences alongside the extraction of respiratory signals. Using navigator echoes or the k-space data (DC signal), these signals can be extracted to reconstruct 3D pulmonary images at different breathing phases. One example is the study published by Weick et al. (2013) [19], in which free-breathing respiratory variations were retrospectively tracked by the DC signal using a Cartesian 3D FLASH sequence, which fills the k-space data in a grid-like manner (sequential filling of the lines of data in the three dimensions). Based on the DC signal magnitude and after setting some threshold values, gating windows were defined, enabling the reconstruction of 3D images at different respiratory phases [19]. This is depicted in Figure 3.

Another recent study introduced XD-GRASP (“eXtra Dimension Golden-angle Radial Sparse Parallel”), a technique in which the k-space data, acquired continuously during free-breathing following a radial trajectory, were grouped into different breathing stages, from expiration to inspiration, by means of the respiratory motion waveform derived from the DC signal. This approach removed blurring and resolved breathing motion, reconstructing 3D images at the grouped stages [30].

The efficacy of the XD-GRASP technique was tested for the simultaneous assessment of lung anatomy and pulmonary ventilation [16]. After reconstructing motion-resolved images at several timepoints, semiautomatic lung segmentations were performed, from which lung volumes were computed. These were employed for calculating tidal volumes (TV), residual volumes (RV), total lung capacities (TLC) and FEV1/FVC ratios (i.e., forced expiratory volume during the first second divided by the forced vital capacity), which achieved good correlations with equivalent spirometry measurements in the same subjects [16].

##### Three-Dimensional Ultrashort Echo-Time (UTE) MRI

Three-dimensional ultrashort echo-time (UTE) sequences using radial k-space sampling were widely explored in pulmonary MRI. Unlike grid-based sampling, k-space data are filled along lines radiating from the k-space center, preserving overall image structure and contrast. UTE sequences can capture signals with very short T2* relaxation times, since the radial trajectories allow to employ significantly short echo times [40]. As a result, they offer an increased signal-to-noise ratio (SNR) and thus better detail of the lung parenchyma. Moreover, they are highly robust to cardiac and respiratory motion, in contrast to Cartesian acquisition schemes [15,25].

Johnson et al. (2013) created an implementation of UTE using a 3T clinical scanner without any hardware modifications [41]. This method proposed three strategies to optimize SNR and image quality of 3D radial UTE pulmonary acquisitions:

1. Limited field of view excitation: This minimizes motion artifacts and improves visualization of areas of interest.
2. Variable density readouts: This involves designing gradient waveforms that adjust sampling density in k-space to improve SNR, especially in short T2* species.
3. Radial oversampling: More points are collected, which enhances image quality and reduces blurring.

This method by Johnson et al. (2013) was commonly employed and referred to in studies using pulmonary UTE [15,25,32,34,41]. It was carried out equivalently in 1.5T systems with similar results [25].

An image-based self-gating approach with a 3D UTE acquisition was later investigated [25]. Here, the navigator-like respiratory signal was obtained from low resolution sliding-window images by extracting the displacement of the lung-liver interface. Similarly to previous studies, some thresholds were determined over the signal magnitude to reconstruct up to eight different stages of the breathing cycle [25].

Another novel technique utilized DC signals in 3D radial UTE MRI to acquire respiratory motion waveforms in neonatal pulmonary imaging [15]. The DC signal was also employed for tracking and discarding MR data corrupted by bulk motion. This technique produced high-resolution images at inspiration and expiration, with reduced respiratory motion blurring. However, it resulted in lower SNR, due to the narrower gating windows used [15].

Semiautomatic lung segmentations were performed on the acquired 3D UTE images to estimate tidal volumes from inspiration–expiration differences. When correlated with spirometry, MRI-derived tidal volumes were slightly smaller. Thus, the study concluded that while the accuracy of this method still needs improvement, it holds potential for assessing lung volumes and function [15].

A different study estimated breathing motion from low resolution, dynamic 3D self-navigating images reconstructed from radial UTE central k-space data [32]. Using these, two strategies were proposed for motion-compensated pulmonary imaging. The first one reconstructed images of single breathing stages after applying lower weights to data from the other phases. The second strategy reconstructed images at multiple breathing stages, by sorting the MRI data into the different phases and optimizing image quality by enforcing spatial and motion sparsity (i.e., emphasizing the most significant features in the image while ignoring redundant information). These methods were successful in the presence of complex breathing behaviors. Both strategies achieved improved quality, motion-compensated images in healthy volunteers and patients [32].

Oxygen-enhanced lung MRI based on 3D radial UTE acquisitions was also investigated [33]. The respiratory signal was, as well, derived from the k-space data and sorted to reconstruct pulmonary images at several breathing phases, following the XD-GRASP principles [30]. This approach provided better detail of the diaphragm’s sharp edge and lung vessels [33].

Deep learning strategies combined with UTE acquisitions were also investigated for acquiring 3D pulmonary MRI images [34]. Specifically, a self-supervised XD-MBDL (“eXtra Dimension, Model Based Deep Learning”) architecture was developed, in which 3D images of a user-selected respiratory phase were reconstructed by combining respiratory states from free-breathing 3D UTE acquisitions. Results showed that this method enhances image quality in terms of apparent SNR and contrast-to-noise ratio (CNR) [34].

#### 3.1.3. Four-Dimensional MRI

For a more comprehensive assessment of respiratory function, capturing the entire breathing cycle during free-breathing rather than specific breathing stages can be essential. To address this need, so-called “4D pulmonary images” were reconstructed, achieving continuous 3D images of the chest motion along a complete cycle.

Various approaches relied on manifold learning, a method that reduces high-dimensional data to a lower-dimensional representation that still preserves the essential information. This technique was employed to extract the respiratory waveform from dynamic 2D slices [29]. Specifically, Laplacian eigenmaps (i.e., neighborhood graphs approximating the manifold on which the data lie) were applied for estimating the breathing stage (low-dimensional representation). Variations between neighboring slices are gradual in both space and time, and slices from different acquisitions but belonging to the same breathing stage are similar. As a result, similar images are closely positioned in the low-dimensional space, and since breathing accounts for the main variation in the images, this information is captured in the first few lower dimensions. This method was correlated with a ground truth signal based on diaphragm tracking, yielding statistically significant correlations up to 0.99 [29].

Similarly, another approach used 2D dynamic MRI acquisitions across all respiratory states at sequential coronal slices of the thorax. These images were “embedded” into a low dimensional space and aligned based on similarity, allowing the reconstruction of 3D high-resolution volumes for all respiratory states [35].

Manifold learning was also applied to k-space data acquired using a radial golden-angle trajectory. Here, k-space profiles were grouped and embedded in a low-dimensional space, so that profiles representing similar phases of the respiratory cycle were close to each other. This enabled the reconstruction of images at different breathing phases by combining nearby radial profiles [31].

A different strategy for 4D MRI was proposed by Tong et al. (2017) [36]. After acquiring continuous MR slices of the torso over multiple respiratory cycles, a graph-based method was developed in which each slice was treated as a node. Connections were drawn between neighboring slices, representing spatial similarity, and were weighted based on the likelihood of being from the same respiratory phase. By finding optimal paths in the graph, 3D images for each respiratory phase were reconstructed. Qualitative evaluation showed good spatial and temporal continuity of the reconstructed images. Quantitative analysis revealed smooth pleural areas and mean volume differences at inspiration and expiration of around 3% when validated against breath-hold acquisitions at the same respiratory stages [36].

Recently, a novel method was developed based on the concept of “optical flux” [37]. Similarly to previous studies, dynamic images of sagittal slices were acquired over several breathing cycles. The respiratory signal for each sagittal location was extracted by estimating the motion vector field of the thoracic region by analyzing consecutive images in time. Motion vectors point outwards during inspiration. Then, this “outgoingness” decreases and finally reverses at expiration, where motion vectors point inwards due to the inward movement of the diaphragm and chest wall. In this way, the “optical flux” was described as the net flux, or net “outgoingness” of the motion vector field going into or out of the thoracic region, and characterizes the respiratory signal with accuracy. Subsequently, abnormal cycles were filtered from the respiratory waveform, considering several measurements such as inspiration and expiration volumes, and amount of peaks and valleys in the curve and distances between them [37]. As a result, a representative breathing cycle for each slice was created using a cosine model, and the cycles from the different slices were joined together to form a final 4D volume, encompassing all phases in a single respiratory cycle with great spatio-temporal continuity [42].

A combination of the graph-based and “optical flux” approaches was also developed [36,37,38]. After acquiring dynamic MRI for each sagittal location over various breathing cycles, the graph-based optimization technique was used to build an optimal 4D image, and automatically labelled end-expiration and end-inspiration phases by analyzing the optical flow velocity information within a region of interest in the diaphragm [38]. This automation represented a significant improvement over the technique used by Tong et al. (2017), where this step was carried out manually to delimit each breathing period [36].

The previous three methods were tested in children with TIS, a condition characterized by chest wall deformities that affect lung function and development. These patients usually present difficulties following breathing instructions or using external tracking devices [38]. Therefore, these studies showed good performance even with complex breathing patterns.

A comprehensive system was recently developed to evaluate thoracic dynamics in TIS patients, named “QdMRI” [43]. It encompasses some of the techniques described above. First, a dynamic MR acquisition with a True-FISP sequence is performed at several locations across the chest during free-breathing, allowing the reconstruction of a 4D image using the optical flux algorithm [37]. Next, a U-Net is employed to segment each lung. Finally, the system retrieves the lung volumes at end-expiration and end-inspiration, as well as at other points of the breathing cycle. In addition, signal intensity was also measured for the evaluation of lung parenchyma aeration [44]. This system has enabled the detection of significant changes in lung volumes in TIS patients before and after surgery [45,46].

## 4. Discussion

The acquisition of chest MR images at different phases of the respiratory cycle is essential in the study of ribcage morphology and pulmonary pathoanatomy in spinal and chest deformities. It presents multiple challenges that have been addressed with a variety of strategies.

Dynamic MRI has been a fundamental technique for the analysis of mechanical and volumetric changes during the breathing cycle. Advances in fast acquisition methods significantly enhanced visualization with high temporal and spatial resolution, improving the evaluation of pathologies such as scoliosis or TIS, which cause significant thoracic deformities [8]. The introduction of 2D and 3D radial UTE MRI improved lung examinations due to its robustness to cardiac and breathing motion and its ability to capture the rapidly decaying signal of the lung parenchyma, increasing SNR and therefore image quality [25,32]. The use of the DC signal (i.e., k-space center signal) was widely exploited in this kind of acquisition to reconstruct pulmonary images at different respiratory stages during free-breathing. Moreover, various techniques, such as manifold learning, graph-based strategies and optical flux, showed promise for reconstructing 4D pulmonary images with high diagnostic value in complex conditions.

When evaluating thoracic volumes and pulmonary function, moderate to good correlations were found between MRI measurements and standard respiratory tests in healthy individuals. Few studies evaluated chest wall motion, diaphragm mechanics and lung volumes in patients with adolescent idiopathic scoliosis or thoracic deformities. Both studies that included AIS patients showed significant respiratory restrictions in these patients when compared to normal controls [26,28]. However, further investigation into these lines should be carried out in the future.

Reconstruction of motion-resolved pulmonary MRI has multiple benefits and clinical implications. First, most of these techniques allow image acquisitions under free-breathing conditions, addressing the challenges posed by patient populations with limited ability to follow instructions or perform sustained breath holds, such as children or individuals with severe respiratory impairments. Moreover, the use of MRI instead of modalities that employ ionizing radiation (e.g., CT) reduces the life-time risk of cancer development, especially in pediatric patients or in conditions requiring a longitudinal evaluation of disease progression. In addition, MRI examination provides a more comprehensive anatomical and functional evaluation of the respiratory system in patients with thoracic deformities. While standard pulmonary function tests remain essential for assessing global respiratory function accurately, MRI adds complementary value by revealing local changes in lung volumes and chest wall movements that cannot be reflected by spirometry alone, providing more targeted information for surgical decision-making.

The recent advancements aimed at reconstructing 4D images must be highlighted, since these approaches combine the high spatial and temporal resolution of 2D dynamic imaging with the volumetric perspective of 3D data, enabling a more detailed and accurate assessment of respiratory mechanics [29,35,36,37,38]. By calculating lung volumes at inspiration and expiration stages, it was possible to derive multiple pulmonary function parameters, such as TV, VC, RV, etc. Moreover, the graph-based and optical flux approaches, as well as Sun et al. (2022)’s method for automatic labelling of end-inspiratory and expiratory phases, that combined notions from the previous two, were shown to work successfully in pediatric population with severe chest deformities [36,37,38].

Some of the studies involving free-breathing acquisitions assumed that the images reflected normal tidal breathing. Therefore, the performance of their strategies on irregular breathing patterns still remains unclear [25,38]. However, some authors did address this aspect, by applying the DC signal for discarding bulk motion, filtering abnormal breathing cycles using the optical flux information or using dynamic 3D self-navigating images to compensate for the irregular breathing patterns [15,32,37].

In dynamic MRI investigations, when employing fast 3D sequences, the required temporal resolution is usually achieved at the expense of spatial resolution. Two-dimensional acquisitions can attain a better image quality, but do not provide a comprehensive visualization of the entire three-dimensional thoracic morphology and shape [36]. This could make them less suitable for evaluating chest wall motion in patients with scoliosis or chest wall deformities, since these are complex 3D deformities.

In the reconstruction of 4D MRI images, computational time is an important consideration. Including more timepoints, which may be useful for the visualization of irregular respiratory behaviors, can increase this computational burden [16]. Despite this, some of the studies reported total reconstruction times of just a few minutes [15,34,37]. Additionally, many of these strategies were not fully automated. Some required manual identification of anatomical structures or regions of interest, manual selection of respiratory stages to delimit different breathing cycles or manual calculation of diameters for the analysis of chest motions [26,32,36,38].

Few studies focusing on motion-resolving techniques in chest MRI extended or validated their findings in the evaluation of thoracic volumes and respiratory function, as most research focused on obtaining high-quality anatomical images. Among the investigations that explored these applications, even fewer have investigated patients with scoliosis or other chest deformities [15,16]. Therefore, it is unsure that some of the techniques in this overview will work correctly in patients with thoracic deformities. Notably, systems such as QdMRI represent a significant advancement by enabling the acquisition of high-quality 4D images and the introduction of image-processing pipelines for evaluating thoracic volumes and ventilation, specifically in patients with thoracic deformities. Furthermore, these approaches also support treatment decisions and planning.

The papers included in this overview generally demonstrate high quality, most of them published in moderate to high-impact journals. Overall, the studies employed robust methodologies, usually supported by well-articulated theoretical frameworks. Both quantitative and qualitative approaches were often used to assess the outcomes of the different strategies, ensuring a comprehensive evaluation of their effectiveness. One of the main limitations across the studies was the lack of diversity in the populations examined, which could impact the generalizability of the findings. Nevertheless, many authors acknowledged these constraints.

### Future Directions

Continued research and development in these areas are essential for improving the diagnostic and monitoring possibilities for patients with spinal or chest deformities. While MRI is widely used for multiple purposes, its application for detailed respiratory evaluation is not yet a routine practice, despite its diagnostic quality and current acquisition times [14]. Automating the strategies described along this paper as much as possible could help accomplish this. In addition, training clinical staff in interpreting the images and performing the required measurements would be essential.

As well, further studies are still necessary to validate the efficacy and reliability of MRI for evaluating thoracic motion and respiratory function. A key consideration is that standard pulmonary function tests (e.g., spirometry) are often performed with the patient seated, while MRI is conducted in a supine position. This difference in positioning can affect the evaluation of respiratory function [7,8]. Hence, understanding how positioning influences measurements and translating MRI results into reliable assessments can be crucial. Earlier studies already anticipated moderate to strong correlations between chest wall measurements in supine MRI and spirometry data in seated position, but this should be further explored in patients with thoracic deformities [8,16].

## 5. Conclusions

This overview described various techniques for acquiring motion-resolved chest images in MRI, including 2D and 3D images at specific stages of the breathing cycle and 4D images, consisting of 3D images covering the entire breathing cycle. In addition, some of these techniques were validated both in healthy subjects and in patients with spinal and/or thoracic deformities by demonstrating their potential for assessing respiratory mechanics and function.

In summary, all the developed strategies can be crucial for performing an accurate evaluation of the impact of rib cage deformities in pulmonary function, not only by addressing the intrinsic challenges of chest MRI that significantly impact image quality, but also by facilitating the imaging process through free-breathing approaches. Moreover, the utility of MRI in avoiding the risks associated with ionizing radiation is demonstrated. However, further research is needed to test and validate such strategies in this context.

## Figures and Tables

**Table 2:** Image types produced by the articles included in this overview.

Image Type	Advantages	Disadvantages
2D	High spatial resolution. High temporal resolution in dynamic acquisitions.	Visualization limited to specific slices.
3D	Visualization of complete chest volumes.	In dynamic acquisitions, temporal resolution is achieved at the expense of spatial resolution.
4D	Visualization of complete chest volumes with high spatio-temporal resolution.	Reconstruction requires advanced techniques with high computational burden.

## Appendix A

**Table A1:** Summary of the papers described in this overview. Studies marked with an asterisk (*) assessed chest wall motion, lung volumes and/or pulmonary function from the acquired images.

Year	Study	Strategy	Outcome/s	Strengths	Limitations	MRI Respiratory Function Parameters
2000	Kondo et al. [21] *	2D dynamic MRI	2D dynamic MRI at specific slices	Good spatial resolution	Patient collaboration is required for performing deep-breathings and breath holds. Manual calculation of distances for the analysis of chest wall motion	Chest wall motion, diaphragm motion and cross sectional areas (at acquired 2d slices)
2004	Plathow et al. [8] *					
	Kotani et al. [28] *					
2006	Chu et al. [26] *					
2007	Chu et al. [27] *					
2012	Wachinger et al. [29]	Manifold learning in 2D dynamic MRI	4D chest MRI	Fully automatic. Good correlation with diaphragm tracking	Manifold learning did not provide a direct differentiation of inspiration and expiration stages	Chest wall motion, diaphragm motion, cross sectional areas (at any slice) and lung volumes for estimation of TC, VC, RV, etc. (see Table 1).
2013	Baumgartner et al. [35]	Manifold learning in 2D dynamic MRI	4D chest MRI	Good spatial resolution in the coronal plane	Thick slices	
	Weick et al. [19]	Breathing motion estimation from DC signal in Cartesian 3D FLASH Sequence	3D chest MRI at expiration, inspiration and intermediate stages	Retrospective self-gating without the need for patient cooperation (free-breathing)	Longer total acquisition times as multiple measurements were needed to ensure enough data for reconstruction	
2016	Feng et al. [30]	XD-GRASP reconstruction	3D chest MRI at a variable number of breathing stages	Allows for reconstructing additional motion-state dimensions	Challenging selection of the appropriate number of respiratory motion stages: too few may limit motion depiction, while too many can lead to increased aliasing artifacts	
	Tibiletti et al. [25]	Image-based self-gating with 3D UTE imaging	3D chest MRI at up to 8 breathing stages	Image-based self-gating technique demonstrated significantly better sharpness and diaphragm excursion compared to DC-based self-gating	Maximal temporal resolution is limited and may not be enough to detect rapidly changing irregular breathing patterns	
2017	Chen et al. [31]	Manifold learning in k-space data	2D and 3D chest MRI at as many respiratory positions as the number k-space profiles (2-D) or the number of stacks of k-space profiles (3-D) acquired	Handles both 2D and 3D scenarios	The effectiveness of the method could decrease if there are insufficient profiles at similar respiratory positions, leading to poor reconstruction quality	
	Tong et al. [36]	Graph-based reconstruction from 2D dynamic MRI	4D chest MRI	Good spatial and temporal resolution. Tested in patients with chest deformities	Requires manual selection of respiratory stages to delimit different breathing cycles	
	Higano et al. [15] *	Breathing motion estimation from DC signal in 3D radial UTE acquisition	3D chest MRI at end-expiration, near end-expiration, inspiration and near-end inspiration	Removal of bulk motion from DC signal. Short reconstruction times	Accuracy of MRI-measured respiratory rates in neonates needs to be improved	
2018	Jiang et al. [32]	3D Dynamic image navigator from 3D UTE data	3D chest MRI at a single stage or 4D chest MRI	Robust to irregular breathing patterns	Requires manual identification of the diaphragm. Long reconstruction time using CPU	
2019	Feng et al. [16] *	XD-GRASP with 3D radial UTE acquisition	4D chest MRI	Improved image quality, detailed anatomical visualization	Half-spoke acquisition technique to reduce scan time, which affects motion detection and scan efficiency	
2021	Hao et al. [37]	Optical flux-based reconstruction from 2D dynamic MRI	4D chest MRI	Good spatial and temporal resolution. Tested in patients with chest deformities. Removal of abnormal breathing cycles. Short reconstruction time. Fully automatic	The cosine function may not be fully suitable to model a normal breathing cycle, as inspiration and expiration phases are not identical in length	
2022	Sun et al. [38]	Graph-based reconstruction + optical flux for automatic labelling of expiration and inspiration stages	4D chest MRI	Good spatial and temporal resolution. Tested in patients with chest deformities	Requires manual identification of region of region of interest at the diaphragm	
	Xu et al. [33]	Oxygen-enhanced lung MRI on 3D UTE acquisition	3D chest MRI at 24 breathing stages (4 were used for final evaluation)	Increased detail of diaphragm’s edge and lung vessels.	Reduced image quality due to under-sampling, to reduce scan time	
2023	Miller et al. [34]	XD-MBDL architecture for 3D UTE MRI	3D chest MRI at user-selected stage	Short reconstruction time	Lack of a ground truth may challenge the evaluation of image quality and anatomy accuracy	
